# DeepIR-Pred: identification of insulin receptors using metaheuristic optimization of biologically informed multi-view features with deep recurrent learning

**DOI:** 10.1093/bib/bbag523

**Published:** 2026-09-28

**Authors:** Matee Ullah, Zhen Li, Yixiao Zhai, Changwei Lv, Kashif Ahmad Khan, Quan Zou, Xu Tan

**Affiliations:** School of Artificial Intelligence, Shenzhen University of Information Technology, Shenzhen 518172, China; School of Life Science and Technology, University of Electronic Science and Technology of China, Chengdu, Sichuan 610054, China; School of Artificial Intelligence, Shenzhen University of Information Technology, Shenzhen 518172, China; School of Artificial Intelligence, Shenzhen University of Information Technology, Shenzhen 518172, China; School of Mechanical Engineering, Tongji University, Shanghai 200092, China; School of Artificial Intelligence, Shenzhen University of Information Technology, Shenzhen 518172, China; Department of Computer Science, Abdul Wali Khan University Mardan, Mardan 23200, Pakistan; Institute of Fundamental and Frontier Sciences, University of Electronic Science and Technology of China, Chengdu, Sichuan 610054, China; Yangtze Delta Region Institute (Quzhou), University of Electronic Science and Technology of China, Quzhou, Zhejiang 324003, China; School of Artificial Intelligence, Shenzhen University of Information Technology, Shenzhen 518172, China

**Keywords:** insulin receptors, pLM embeddings, image-based features, SBWOA, deep learning, BiGRU

## Abstract

The insulin receptor (IR) proteins play a central role in cellular growth and metabolic regulation. Disruption of IRs is linked to several fatal neurodegenerative and cancer-like diseases. Wet-lab experiments are time-consuming, costly, and resource-intensive. Therefore, accurately predicting IR proteins using computational methods is critical for understanding the IR-associated biological mechanism and advancing therapeutic research. In this work, we propose a biologically informed deep learning method, DeepIR-Pred, which is the first rigorous model to accurately identify IR proteins. To ensure model robustness, two rigorously curated non-redundant sequence-based training and testing datasets (IR2476 and IR274) were first constructed. A newly proposed transformed image-based feature descriptor [Position-Specific Scoring Matrix (PSSM)–rotation-invariant co-occurrence among adjacent local binary patterns], derived from PSSMs, was integrated with biologically informed qualitative characteristics and protein language model embeddings (Evolutionary Scale Modeling 2 and ProtTrans-T5). Next, an enhanced metaheuristic-based spatial bound whale optimization algorithm was employed to reduce and select the most discriminative features. Finally, DeepIR-Pred was learned using a deep recurrent neural network (Bidirectional Gated Recurrent Unit) architecture. Extensive experimental results, using 10-fold cross-validation and independent testing, demonstrated the significant performance of the DeepIR-Pred in terms of both robustness and generalization capability on unseen samples. Furthermore, interpretability analysis provided insights into feature contributions and discriminative characteristics associated with IR proteins, while the uniform manifold approximation and projection (UMAP) analysis highlighted improved class separability. Collectively, DeepIR-Pred paves the way for researchers to investigate computational-based methods for IR proteins in depth and unlock new potential in therapeutic discovery. All data and source codes for DeepIR-Pred are available at https://github.com/MateeullahKhan/DeepIR-Pred for academic use.

## Introduction

Insulin signaling is an essential biological process that plays a critical role in regulating glucose utilization, cellular growth, and energy homeostasis in multicellular organisms. The insulin receptor (IR), a transmembrane tyrosine kinase, central to insulin signaling, mediates cellular response to insulin in various tissues including muscle, liver, adipose tissue, and the central nervous system underscoring its central role in cellular growth and metabolic regulation [1, 2]. Upon activation, the IR triggers a cascade of phosphorylation-driven signaling, mainly through the Ras–MAPK and IRS–PI3K–Akt pathways. This controls numerous anabolic processes including glucose uptake, suppression of gluconeogenesis, and glycogen production. Additionally, the IR also enhances lipid synthesis while suppressing lipolytic activity, thereby aiding energy storage during nutrient-rich conditions [3]. Moreover, ﻿in the central nervous system, the IR plays a pivotal role in cognitive functions particularly involving memory and learning. Disruption of the IR signaling pathway is associated with several neurodegenerative diseases including the fatal Alzheimer’s disease, highlighting its physiological importance [4, 5]. In oncology, IR is known as a critical therapeutic target. Aberrant signaling is linked to cancer ﻿progression and targeted therapies like tyrosine kinase inhibitors are under investigation to intervene in these oncogenic pathways.

Given the critical role of the IR in numerous human-related illnesses, scientists and researchers have been motivated to pursue further investigations. The identification and study of IR have employed various experimental approaches, including radioligand binding assays [6], western blotting [7], immunohistochemistry [8], immunofluorescence [9], flow cytometry [10], and affinity chromatography [11]. These experimental approaches have evolved over time and made it possible to learn about the IR shape, dynamics, and various important biological processes. However, these experimental techniques still have several important limitations. They require a significant amount of resources and expertise. Furthermore, the successful implementation of these techniques often requires high-quality biological specimens, a requirement that can be challenging to obtain for complex cellular environments, tissues, or experimental conditions. In addition, they are time-consuming and costly which further limits these experimental techniques for large-scale IR identification.

During the last decades, computational-based identification has provided an alternative way to these time-consuming, resource-intensive, and costly experimental techniques, which accurately analyze and identify a range of biological proteins more rapidly with low operational cost [12–14]. Therefore, there is also a growing demand for computer intelligence-based methods for efficiently predicting IRs. Various feature descriptors have been proposed to effectively extract meaningful information such as evolutionary information and latent statistical patterns [15], physicochemical information [16], and structure-based information [17] from protein sequences. They require minimal effort with low resources. Additionally, deep learning-based models have become popular in recent years, achieving high performance across various research fields [18–21]. Therefore, ﻿to accurately predict the IR, deep learning models are the best choice as they can easily accommodate the rapidly growing biological data, achieving high accuracy with unprecedented speed that wet-lab experimental techniques alone cannot achieve effectively. While deep learning models have revolutionized protein science, their application to predicting the IR remains underexplored. To fill this gap, there is an urgent need to develop a deep learning model specifically targeting IRs.

In this study, we propose the first deep recurrent-based computational model to predict IRs from non-IRs. To effectively predict IRs, following are the five main contributions of this study: (i) construction of new benchmark training and testing IRs datasets; (ii) we propose a new transformed image-based evolutionary feature extraction method, PSSM_RICLBP, integrated with qualitative characteristics (QLC) features, Evolutionary Scale Modeling 2 (ESM2), and ProtT5 embeddings; (iii) a newly developed metaheuristic-based feature selection (FS) algorithm was introduced to optimally select best features and reduce the dimension of large multi-view protein vectors; (iv) a new deep bidirectional recurrent neural network (RNN) was specifically designed to effectively predict IR proteins and; (v) the generalization ability of the model was tested using an independent test dataset. Based on the above-mentioned pipeline and contribution, a powerful deep learning model, DeepIR-Pred, was implemented. Experimental results on the training and testing datasets further verify the model’s efficiency.

## Datasets and methods

### Dataset

A more reliable and stringent predictive model can only be developed when it is trained upon vigilantly created and collated datasets that are rigorous, comprehensive, and reliable. Therefore, in this study, we constructed our own protein sequence-based dataset for the IR. The sequence samples were carefully derived from the widely known data source UniProt (https://www.uniprot.org) [22]. The dataset is specifically collected for the problem of binary classification and contains IR sequences as the positive class and non-IR sequences as the negative class. The IR samples were retrieved by querying the keyword “Insulin receptor.” The reviewed filter (Swiss-Prot) was enabled which ensures that only high-quality and manually curated proteins were collected. For the negative class, proteins were derived using the intentionally designed query “Kinase AND Cytoplasm NOT Transmembrane NOT Signal” with retrieval restricted to reviewed only. This ensures that the negative class is biologically meaningful and challenging for the model rather than randomly selected proteins and hence cytoplasmic kinases without signal peptides and transmembrane regions were collected as negatives to ensure functional similarity while avoiding overlap with IRs. A total of 3255 IR and 11 218 non-IR sequences were retrieved.

To ensure the data quality, several preprocessing steps were applied. First, the unknown or non-standard amino acids ﻿B, J, U, Z, X, O were removed to avoid inconsistencies and noise and then sequences with amino acids <50 were filtered out. Next, to mitigate data scarcity and ensure there were enough sequences for training the deep learning model while preserving sufficient functional diversity as well, we employed the CD-HIT [23] tool with 60% similarity threshold to reduce the sequence redundancy. Stricter similarity thresholds were employed as well, which resulted in substantial data reduction, leading to data scarcity, and reduced functional diversity; hence, this threshold was empirically selected to remove highly similar samples. Finally, the IR protein reached 1375 and the non-IR protein reached 3026. Next, we randomly selected 1375 non-IR from the 3026 samples and the final obtained sequences were carefully checked to ensure there were no duplicates selected. We then divided the dataset into training and independent sets. The final training dataset comprises 1238 IR proteins (positive samples) and 1238 non-IR proteins (negative samples), while the independent test dataset consists of 137 positive and 137 negative samples. The trained and independent datasets were named IR2476 and IR274, respectively. Table 1 summarizes the details of both datasets, while the compositional analysis is provided in Supplementary Figure S1 under Supplementary Text S1 of the SI.

**Table 1:** Statistical summary of the datasets.

Protein	Original	Non-redundant	Balanced	IR2476	IR274
IR	3255	1375	1375	1238	137
Non-IR	11 218	3026	1375	1238	137

### Feature representation approaches

#### Representation of insulin receptor sequence as PSSM

The Position-Specific Scoring Matrix (PSSM) is a widely used representation for capturing evolutionary information in protein sequences. PSSM has shown remarkable efficiency across many bioinformatics problems [24–27]. Collectively, these existing studies underscore the significant contribution of evolutionary information to improved predictive modeling.

In this work, protein sequences were represented using their corresponding PSSM profiles. For a given L-length IR sequence, an L × 20 PSSM matrix was obtained, where 20 represents the standard amino acids. Each element, at a given position in the matrix, denotes the residue’s evolutionary mutation probability. A standard mathematical representation of the PSSM matrix P is provided in Equation 1.


(1) \begin{eqnarray*} P={\left[\begin{array}{cccc}{p}_{1,1}& {p}_{1,2}& \cdots & {p}_{1,20}\\{}{p}_{2,1}& {p}_{2,2}& \cdots & {p}_{2,20}\\{}\vdots & \vdots & \vdots & \vdots \\{}{p}_{L,1}& {p}_{L,2}& \cdots & {p}_{L,2}\end{array}\right]}_{L\times 20} \end{eqnarray*}

To address the limitation of PSSM’s variable length, this study employed a novel technique that transforms the PSSM matrix into a 2D image representation and encodes it using an image-based feature encoding strategy. The details are provided in the subsequent section.

Notably, this study employed the local NCBI-BLAST+ [28] program together with the Swiss-Prot database [29, 30] to extract the PSSM profile for each provided sequence.

##### RICLBP-based encoding of image-transformed PSSM

As discussed earlier, the PSSM profile encodes evolutionary conservation information across protein sequences. In its L × 20 transformed image representation, these patterns appear as texture structures, where intensity variations indicate residue conservation and mutation. This representation allows the extraction of spatial structures inherent in the evolutionary profiles. To efficiently capture these patterns, rotation-invariant co-occurrence among adjacent local binary patterns (RICLBP) [31] was employed in this study. Through co-occurrence relationships between neighboring patterns, RICLBP allows the model to effectively capture higher-order spatial dependencies and structured evolutionary variations, enhancing the discriminative power of the feature representation. The step-by-step process is provided below.

1. PSSM Image transformation.

For a given PSSM *P*, its transformed PSSM image is represented as *P_I_* with intensity values between 0 and 255.

2. Local Binary Pattern (LBP) feature computation.

For each pixel in the PSSM image *P_I_*, the LBP code is calculated by comparing the central pixel *p_c_* with its neighboring pixels *p_n_* = {*p_0_, p_1_*, …, *p_p-1_*}:


(2) \begin{eqnarray*} LB{P}_{N,R}=\sum \limits_{n=0}^{N-1}s(d){2}^n,\kern2em d={p}_n-{p}_c \end{eqnarray*}

If the value of the corresponding neighboring pixel is equal to or greater than a given threshold, the function *s(d)* assigns a value of 1, and 0 otherwise:


(3) \begin{eqnarray*} s(d)=\left\{{}_{0,\mathrm{otherwise}}^{1,d\ge 0}\right. \end{eqnarray*}

3. Rotation-invariant transformation.

The LBP codes are further transformed into rotation-invariant uniform LBP to ensure robustness against rotation:


(4) \begin{eqnarray*} LB{P}_{N,R}^{riu2}=\left\{{}_{N+1,\kern3.5em \mathrm{otherwise}}^{\sum_N^{N-1}s(d),\kern1.25em \mathrm{if}\ U\left( LB{P}_{N,R}\right)\le 2}\right. \end{eqnarray*}

where $U\left( LB{P}_{N,R}\right)$ denotes the number of bit transitions. This operator improves robustness by ensuring rotation invariance while reducing dimensionality by aggregating non-uniform patterns.

4. Co-occurrence matrix construction.

To capture spatial relationships among adjacent local patterns, a co-occurrence matrix is constructed for each spatial displacement as follows:


(5) \begin{eqnarray*} C\left(a,b\right)=\sum \limits_{x,y}I\left( LB{P}_{N,R}^{riu2}\left(x,y\right)=a\right)\cdot I\left( LB{P}_{N,R}^{riu2}\left(x+\varDelta x,y+\varDelta y\right)=b\right) \end{eqnarray*}

where $I\left(\cdot \right)$ is the indicator function that yields 1 when the condition holds and 0 otherwise. $LB{P}_{N,R}^{riu2}\left(x,y\right)$ represents the rotation-invariant uniform LBP at position (*x, y*). (∆*x*, ∆*y*) defines the spatial displacement.

Each co-occurrence matrix is then normalized and the final feature vector is obtained by flattening the normalized co-occurrence matrix:


(6)

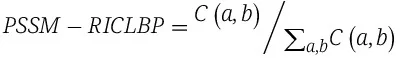



To encode multi-scale spatial patterns, the above process is performed using two parameter settings (1, 2) and (2, 4). For each parameter setting, a fixed-length vector of 136 features is obtained and by concatenating them, we obtain 272D features which serve as the final PSSM-RICLBP feature vector.

#### Qualitative characteristics features

The QLC feature [32] is a global representation method that characterizes amino acid sequences based on their underlying physicochemical properties. It incorporates seven fundamental physicochemical properties of amino acids, including polarity, charge, polarizability, solvent accessibility, hydrophobicity, normalized van der Waals volume, and secondary structure. These properties are divided into three separate classes based on their shared properties. Complete details for each group can be found in Supplementary Table S1 under Supplementary Text S2 in the SI. QLC offers a biologically meaningful representation by transforming complex sequence information into patterns derived from physicochemical properties which makes it well-suited for machine learning approaches.

QLC adopts the composition–transition–distribution framework [33], which allows it to capture complementary information about residue behavior in the sequence. The composition (C) operator measures the proportion of residues belonging to each physicochemical property group, which provides global information about sequence content. The transition (T) operator quantifies the transition frequency between two adjacent residues, thereby encoding local sequence order and variability information. The distribution (D) operator measures the positional spread of residues within the sequence at 25%, 50%, 75%, and 100% intervals. Encoding global composition with local transitions and distribution allows the model to effectively detect structural signals and functional motifs embedded in protein sequences while maintaining computational efficiency and interpretability compared to high-dimensional descriptors. For each provided IR protein sequence, the QLC generates a 147D feature vector with the C, T, and D operators contributing 21D, 21D, and 105D features, respectively.

#### Sequence encoding using protein language models

Protein language models (pLMs) use deep learning architectures, mainly transformers, to encode amino acid sequences into numerical representations by learning contextual relationships between residues. These dense numerical representations are often called embeddings. They are trained on a large-scale amino acid sequence database that learns rich representations, including structural, functional, and evolutionary information. Notably, pLM-based embeddings eradicate the need for computationally expensive multiple sequence alignment (MSA) and manual feature engineering while preserving biologically meaningful information.

In this study, we employed two state-of-the-art pLMs models, including evolutionary scale modeling (ESM) [34] and ProtTrans [35]. Figure 1 graphically explains how the two models were employed to obtain the sequence embeddings, while the details for each model are discussed in the following subsections.

**Figure 1:**
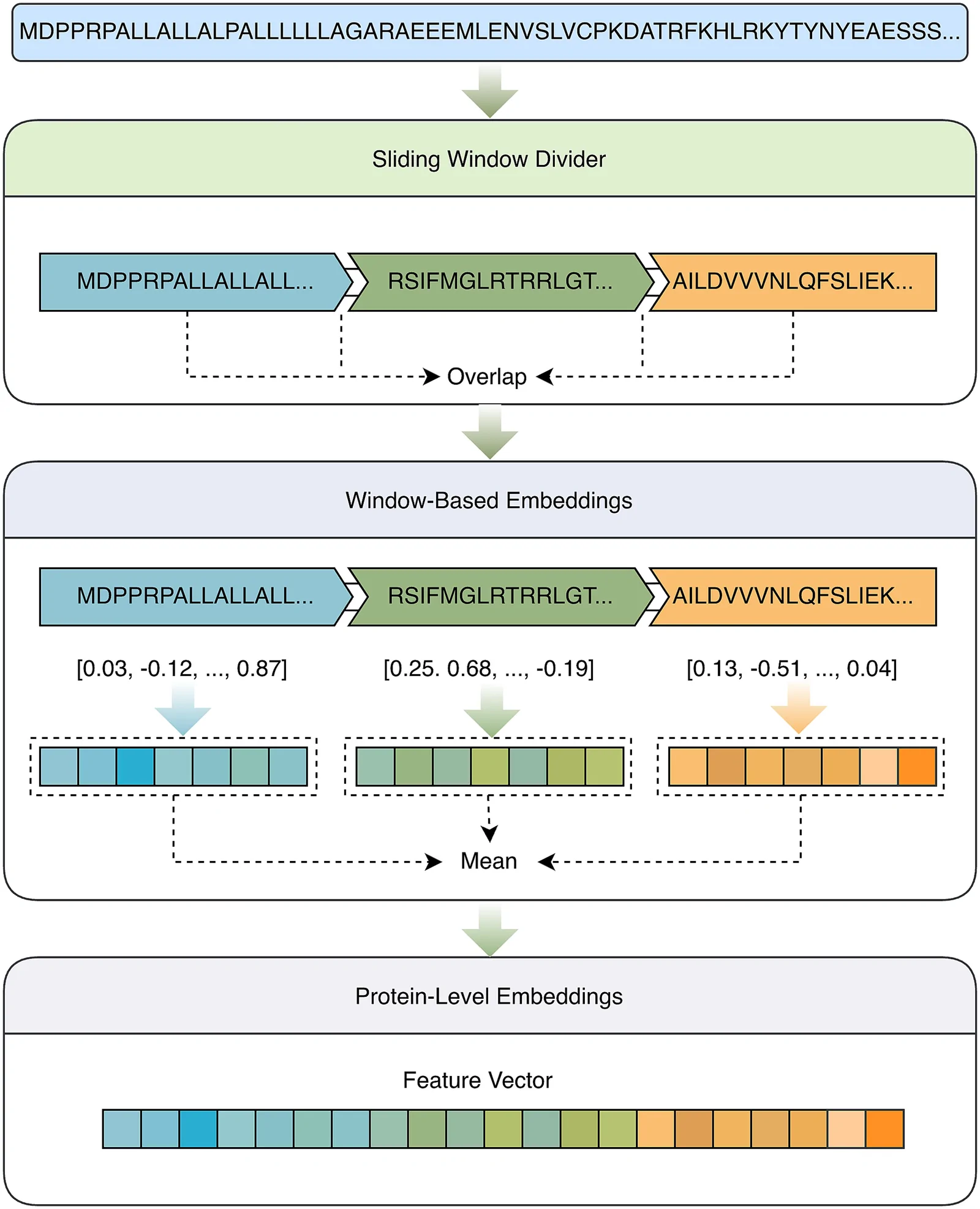
Architecture diagram showing the working mechanism of pLMs in this study.

##### Evolutionary scale modeling

Built upon the Bidirectional Encoder Representations from Transformers architecture, the ESM model incorporates self-attention mechanisms to learn complex residue–residue interactions and long-range dependencies within protein sequences. The model is trained on a large-scale protein database, allowing it to learn complex contextual patterns associated with structural, functional, and evolutionary information.

Several ESM variants have been developed and trained, differing in training data, architectural complexity, and model size. Newer variants, such as ESM2, have further improved the performance of pLMs through the expansion of training data and architectural complexity. As discussed above, ESM models do not require MSA or homologous sequences, which make them suitable for large-scale protein analysis due to their computational efficiency. In this work, we employed the ESM2 model (esm2_t33_650M_UR50D), which has 650 million parameters and was trained on UniRef50 [36]. Protein sequences are first converted into FASTA format and then provided as input to the pretrained ESM2 model to extract sequence embeddings. To ensure the model incorporates the full sequence length while maintaining computational efficiency, sequences exceeding the maximum input length were chunked into a maximum of 1022 residues using a sliding window strategy with an overlap of 256 residues between consecutive chunks. Each chunk was tokenized using the ESM2 tokenizer and input independently to the ESM2 pretrained model, and residue-level embeddings were obtained from the penultimate layer. To retain only sequence representation, tokens were then excluded from the obtained embeddings. For residues that appeared in multiple overlapping regions, their embeddings were averaged to mitigate boundary effects and guarantee consistency. Finally, a global average pooling strategy was employed to obtain fixed-length 1280D embeddings, which served as the final feature vector for the corresponding protein sequence.

##### ProtTrans-T5

ProtTrans-T5 (referred to as ProtT5 in this study) is another state-of-the-art pLM built on the architecture of the Text-to-Text Transfer Transformer (T5) [37], in which protein sequences are treated the same as natural language while amino acid residues are represented as tokens. This approach allows the model to analyze amino acid sequence globally rather than considering local patterns. Like the ESM2, ProtTrans-T5 also relies on a self-attention mechanism, which effectively captures long-range dependencies and complex residue-residue interactions within protein sequences.

In this study, the ProtT5-XL-UniRef50 model was employed to obtain sequence embeddings. Specifically, protein sequences were first preprocessed to map unambiguous characters (U, Z, O, B) to X. To handle and process the full-length sequences, sequences exceeding the maximum length were chunked into a length of 512 tokens using a sliding window strategy with an overlap of 128 residues. Each chunk was then processed independently by ProtT5 to obtain per-residue embeddings, and embeddings for those residues that appeared in overlapping regions were averaged. Finally, a global average pooling strategy was adopted to generate a fixed-length 1024D embedding (feature vector) for each protein sequence.

### Feature selection using metaheuristic-based approach

FS plays a vital role in high-dimensional feature space by selecting the most informative and non-redundant features by removing redundant and irrelevant features. In bioinformatics, the search space expands exponentially with the number of features, making optimal search computationally infeasible. To address this issue, metaheuristic optimization algorithms are widely utilized due to their ability to effectively explore large and complex search spaces. Among them, an enhanced variant of the Whale Optimization Algorithm (WOA) [38] called the Spatial Bound Whale Optimization Algorithm (SBWOA) [39] has gained popularity. The WOA emulates the bubble-net hunting approach of humpback whales. WOA updates the candidate solutions through encircling prey, spiral movement, and random exploration; however, it fails to control the size of the selected feature subset, which can easily lead to suboptimal solutions with either excessively large or insufficient features. To address this issue, SBWOA employs a spatial bounding approach that vigorously restricts the number of selected features within predefined bounds. The step-by-step process of SBOWA is as follows:

1. Initialization.

Let ${X}_i={\mathbb{R}}^D$ be the population of whales (candidate solutions), where *i=1,2,3,...,N*, *D*represents the total number of features. Each whale corresponds to a binary vector indicating the inclusion or exclusion of features:


(7) \begin{eqnarray*} {X}_i=\left\{{x}_1,{x}_2,...,{x}_D\right\},\kern0.75em {\mathrm{x}}_j=\left\{0,1\right\} \end{eqnarray*}

2. Encircling prey.

﻿The whales identify the target prey as the best candidate position, and update their position *X*toward the optimal solution ${X}_{gb}$ as follows:


(8) \begin{eqnarray*} {X}^{t+1}={X}_{gb}^{(t)}-A\cdot \left|C\cdot{X}_{gb}^{(t)}-{X}^{(t)}\right| \end{eqnarray*}

where *t* represents the current iteration. $A=2a{r}_1-a$ and $C=2{r}_2$ are the two coefficient vectors with ${r}_1$ and ${r}_2$ representing two random vectors in intervals [0, 1] and *a* denotes a parameter that decreases linearly from 2 to 0.

3. Bubble-net attacking.

The whales also attack their target prey via the bubble-net attacking method, where they utilize shrinking encircling and spiral updating strategies. Reducing the value of parameter *a* in $A=2a{r}_1-a$ achieves the shrinking encircling strategy. In the strategy of spiral updating position, the whales also update position *X* in a spiral trajectory as follows:


(9) \begin{eqnarray*} {X}^{\left(t+1\right)}=\left|{X}_{gb}^{(t)}-{X}^{(t)}\right|\cdot{e}^{bl}\cdot \cos \left(2\pi l\right)+{X}_{gb}^{(t)} \end{eqnarray*}

where the constant *b* represents the logarithmic spiral shape and *l* denotes the random vector between the intervals [−1, 1].

4. Searching the prey.

In searching for the prey phase, the movement of the whale is determined by the coefficient vector *A*. When $\left|A\right|>1$, the whale moves farther from a randomly selected whale position ${X}_{rand}$. This strategy allows the exploration of the search space globally and prevents the algorithm from being trapped in the local optima:


(10) \begin{eqnarray*} {X}^{\left(t+1\right)}={X}_{rand}-A\cdot \left|C\cdot{X}_{rand}-{X}^{(t)}\right| \end{eqnarray*}

5. Spatial bound mechanism.

To satisfy the subset size constraints, SBWOA introduces a spatial bounding mechanism to control the number of selected features:


(11) \begin{eqnarray*} {X}^{\left(t+1\right)}\to Bound\left[{X}^{\left(t+1\right)}, LB, UB\right] \end{eqnarray*}

where *Bound* ensures


(12) \begin{eqnarray*} LB\le \sum \limits_{j=1}^d{x}_j\le UB \end{eqnarray*}

$UB=\left({d}_{\mathrm{max}}\cdot D\right)$ and $LB=\left({d}_{\mathrm{min}}\cdot D\right)$ are the maximum and minimum number of allowable features, respectively. *D* represents the size of the original feature set while ${d}_{min}$ and ${d}_{max}$ are the minimum and maximum dimension rates selected via the tournament selection scheme.

In this study, the SBWOA is employed after serially concatenating all the original extracted feature sets, i.e. PSSM-RICLBP+QLC + ESM2 +ProtT5.

### Implementation of deep BiGRU

The Bidirectional Gated Recurrent Unit (BiGRU) is a type of RNN designed to handle sequential data efficiently. It is an extension of the standard gated recurrent unit (GRU) [40]. In contrast to conventional GRUs, which process sequence data in one direction, BiGRUs process the input sequences in both forward and backward directions, allowing the model to capture dependencies bidirectionally. This mechanism of the BiGRU is particularly useful for tasks including protein sequences, natural language processing, or temporal data, where information from prior and subsequent states provides valuable context.

A GRU employs the update and reset gates to control the information flow through the network. Specifically, the update gate controls how much information from the previous state is incorporated into the present state. A larger value of the update gate enables more past state information to be retained. Similarly, the reset gate of the GRU controls the amount of information to be disregarded from the previous state, allowing the model to forget irrelevant information. The update ${z}_t$ and reset ${r}_t$ gates are expressed as:


(13) \begin{eqnarray*} {z}_t=\sigma \left({W}_z\left[{h}_{\left(t-1\right)},{x}_t\right]+{b}_z\right) \end{eqnarray*}

(14) \begin{eqnarray*} {r}_t=\sigma \left({W}_r\left[{h}_{\left(t-1\right)},{x}_t\right]+{b}_r\right) \end{eqnarray*}

Where ${x}_t$ is the input vector at time *t*, ${h}_{\left(t-1\right)}$ is the previous hidden state, ${W}_z$ and ${W}_r$ are the learnable weights, and ${b}_z$ and ${b}_r$ are the bias terms. The final hidden representation ${h}_t^{BiGRU}$ is passed through a dense layer to output the prediction probabilities for IR proteins:


(15) \begin{eqnarray*} y=\sigma \left({W}_y{h}_t^{BiGRU}+{b}_y\right) \end{eqnarray*}

where *σ* denotes the sigmoid activation function as:


(16) \begin{eqnarray*} \sigma (x)=\frac{1}{1+{e}^{-x}} \end{eqnarray*}

### Architecture of DeepIR-Pred

The architecture of the proposed DeepIR-Pred is illustrated in Fig. 2. For each provided input IR sequence, DeepIR-Pred first captures raw feature sets by calling the PSSM-RICLBP, QLC, ESM2, and ProtT5 feature extraction schemes (Feature Extraction in Fig. 2). All four extracted feature sets are concatenated in a serial fashion and further sent as an input into the metaheuristic SBWOA FS algorithm to obtain a low-dimensional optimal feature set (Feature Integration and Selection in Fig. 2). Finally, the low-dimensional optimal feature set is obtained for all the sequences in the IR2476 training dataset and sent into the designed BiGRU network to train DeepIR-Pred for the Target IR proteins (Model Training and Performance evaluation in Fig. 2). For prediction and generalizability, each provided IR sequence that needs to be predicted, is transformed into the low-dimensional optimal feature set using the Feature extraction, Feature integration and selection stages developed during model training. Finally, the trained model is then employed to predict the corresponding IR protein. In Fig. 2, the whole training procedure is represented by solid arrows, while the testing phase is represented by dotted (dashed) arrows.

**Figure 2:**
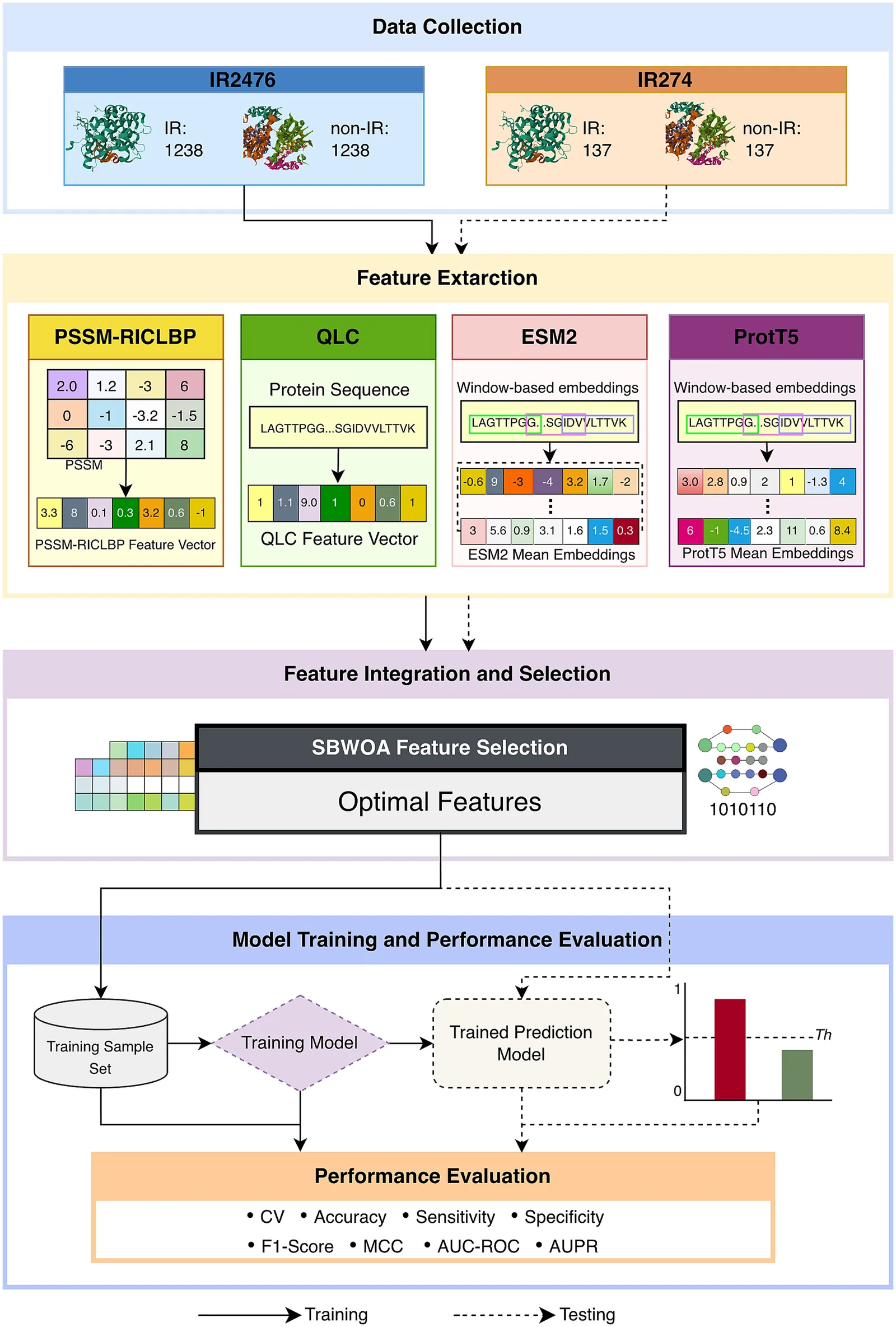
Architecture of the proposed DeepIR-pred model.

### Model evaluation metrics

The predictive performance of DeepIR-Pred was quantified using several widely utilized metrics designed for classification problems. Precisely, Accuracy (Acc), Matthews Correlation Coefficient (MCC), Sensitivity (Sen), Specificity (Spe), and F1-Score were selected to quantify the performance across multiple conditional and joint distributions of true and predicted outputs. The formal definitions along with the mathematical formulas of these metrics are provided in Supplementary Text S3 of the SI. In addition, the DeepIR-Pred was further assessed using the Area Under the Receiver Operating Characteristic Curve (AU-ROC curve or shortly AUC) and Area Under the Precision–Recall Curve (AUPR curve). Both AUC and AUPR provide a comprehensive and threshold-independent assessment of the model’s predictive performance across IR and non-IR samples.

To ensure that DeepIR-Pred performance is robust and to minimize the bias toward the training dataset, the model was validated on unseen data. In this regard, a stratified k-fold cross-validation (CV) strategy was adopted to maintain the class proportions and reduce the risk of overfitting. Besides, to assess the model’s generalization capability on new unseen data, an independent testing strategy, where the independent test set (IR274) was completely excluded and untouched during the training, was implemented as well.

To enable the reproducibility of the proposed model, the detailed software and hardware configurations, and implementation settings used in this study, are provided in Supplementary Text S4 of the SI.

## Results and discussion

### Performance analysis using individual encoding methods

As discussed in the section “Feature representation approaches,” four novel feature encoding schemes (PSSM-RICLBP, QLC, ESM2, and ProtT5) were employed to represent IR protein sequences as feature vectors. This subsection evaluates and compares the performance results of these four feature sets on the benchmark training IR2476 dataset using various state-of-the-art learning models, i.e. deep neural network (DNN), convolutional neural network (CNN), bidirectional long-short term memory (BiLSTM) network, GRU, and BiGRU. The performance of these models on the extracted feature sets was validated using 10-fold CV and the output results are reported in Table 2.

**Table 2:** Performance comparison of individual feature sets using IR2476 training dataset.

Feature	Classifier	Acc (%)	Sen (%)	Spe (%)	F1-Score (%)	MCC	AUC
PSSM-RICLBP	DNN	90.46	90.46	90.46	90.48	0.8100	0.9582
	CNN	88.04	88.61	87.47	88.13	0.7613	0.9511
	BiLSTM	90.47	91.03	89.90	90.52	0.8098	0.9692
	GRU	89.58	89.25	89.90	89.54	0.7921	0.9584
	BiGRU	90.63	89.90	91.35	90.56	0.8131	0.9633
QLC	DNN	87.76	86.02	89.49	87.53	0.7574	0.9483
	CNN	86.38	87.15	85.62	86.48	0.7286	0.9398
	BiLSTM	88.08	88.85	87.31	88.18	0.7596	0.9481
	GRU	87.92	88.04	87.79	87.93	0.7596	0.9492
	BiGRU	88.57	88.93	88.20	88.59	0.7730	0.9494
ESM2	DNN	88.31	86.67	89.97	88.15	0.7672	0.9542
	CNN	90.58	88.45	92.74	90.40	0.8134	0.9710
	BiLSTM	90.74	88.45	93.06	90.56	0.8167	0.9723
	GRU	90.58	88.53	92.66	90.40	0.8133	0.9691
	BiGRU	91.31	90.55	92.09	91.28	0.8273	0.9734
ProtT5	DNN	89.24	87.48	91.03	89.08	0.7860	0.9567
	CNN	90.95	89.66	92.26	90.87	0.8201	0.9712
	BiLSTM	90.87	87.65	93.64	90.37	0.8145	0.9709
	GRU	90.87	88.13	93.52	90.65	0.8193	0.9716
	BiGRU	91.11	88.61	93.64	90.91	0.8239	0.9726

Observing Table 2, first, the proposed handcrafted PSSM-RICLBP achieved better performance than the handcrafted QLC. Specifically, PSSM-RICLBP achieved the best performance on BiGRU with Acc = 90.63%, F1-Score = 90.56%, MCC = 0.8131, and AUC = 0.9633, which provides 2.06%, 1.97%, 4.01%, and 1.39% better performance than the best output model (BiGRU) on QLC. By comparing the deep embeddings of two pLMs, i.e. ESM2 and ProtT5, ESM2 has the highest performance on the BiGRU with Acc, F1-Score, MCC, and AUC of 91.31%, 91.28%, 0.8273, and 0.9734, respectively. Similarly, observing and comparing the results of PSSM-RICLBP with the ESM2 and ProtT5, the proposed PSSM-RICLBP still achieved competitive performance on all the trained learning models. Second, by comparing all the classification models, the BiGRU consistently outperformed the other models, i.e. DNN, CNN, BiLSTM, and GRU, reported in Table 2, in terms of all performance metrics.

To further show the predictive strength of the individual feature sets, threshold-independent assessment was carried out in terms of ROC and PR curves. The plotted results in terms of ROC are illustrated in Fig. 3, while the PR curves are presented in Fig. 4. Both Figs 3A–D and 4A–D show the performance of all the classification models. By carefully observing these figures, we can easily conclude that all the extracted feature sets achieved competitive performance on all the reported classification models, with BiGRU achieving the best performance. In addition, Fig. 5A–D also compares the models in terms of all performance metrics on the four feature sets, which further validates the model performance on these feature sets. Together, these results suggest that the extracted feature sets are vital for effectively discriminating IR proteins from non-IR proteins.

**Figure 3:**
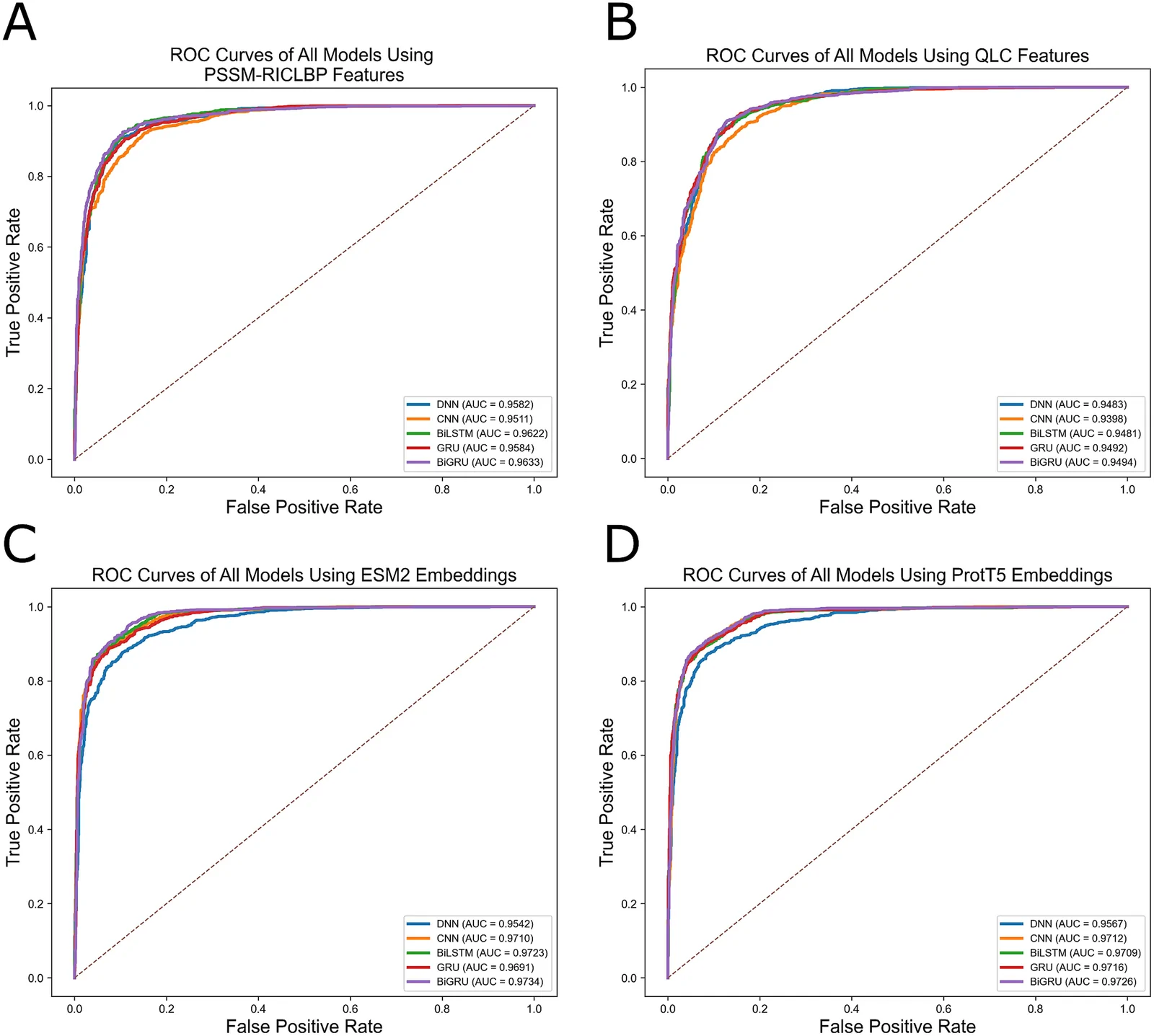
ROC curves of individual features: (A), (B), (C), and (D) show the ROC curves of PSSM-RICLBP, QLC, ESM2, and ProtT5, respectively, on all the reported classification models.

**Figure 4:**
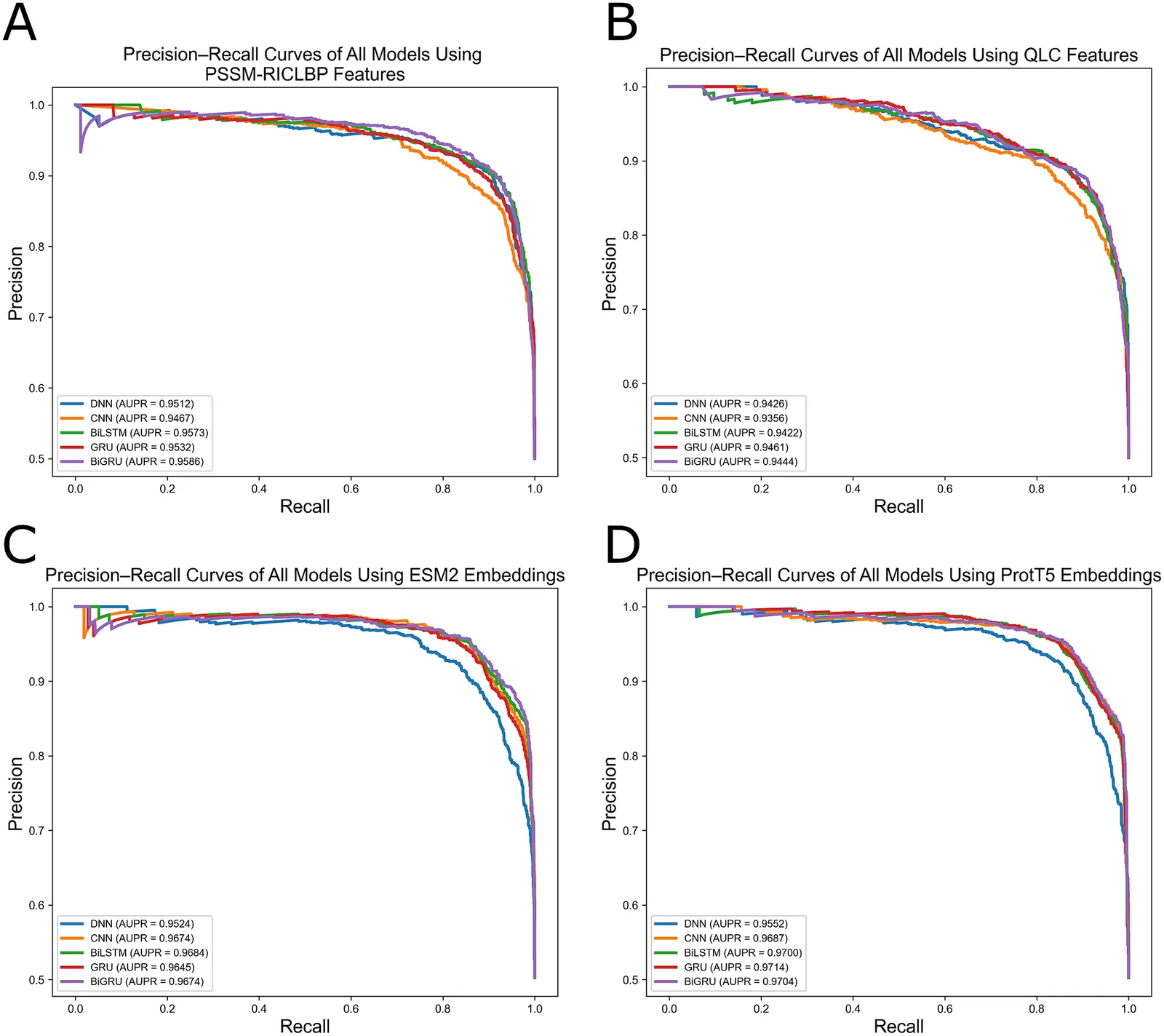
PR curves of individual features: (A), (B), (C), and (D) show the PR curves of PSSM-RICLBP, QLC, ESM2, and ProtT5, respectively, on all the reported classification models.

**Figure 5:**
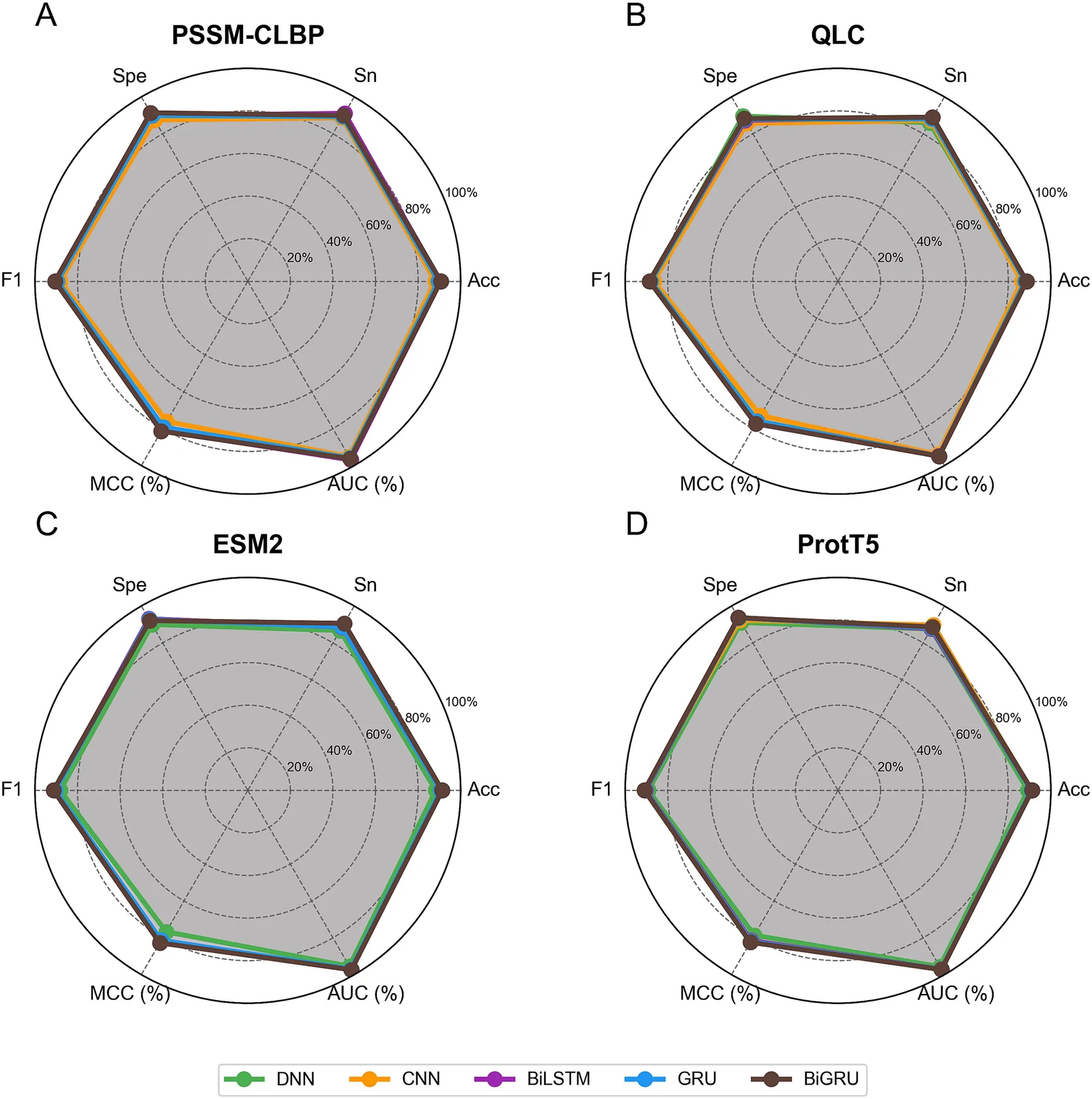
Performance comparison using radar chart: (A), (B), (C), and (D) show models performance on PSSM-RICLBP, QLC, ESM2, and ProtT5, respectively.

### Performance analysis after feature integration

In the previous subsection, we showed the performance of individual features on different classification models. Although they performed well, the individual features still lack meaningful information. Therefore, integrating multiple feature sets can further strengthen the predictive and generalization performance of the model [41–43]. In this section, we integrated features based on their complementary representations, i.e. handcrafted features with pLMs, using simple serial integration strategy and thus, the following three combinations can be obtained:

- PSSM-RICLBP+QLC + ESM2
- PSSM-RICLBP+QLC + ProtT5
- PSSM-RICLBP+QLC + ESM2 + ProtT5

The sign “+” denotes simple serial concatenation. The above three combinations were experimentally assessed using the benchmark training IR2476 dataset. Table 3 reports the output results of these hybrid features.

**Table 3:** Performance comparison of integrated features using IR2476 training dataset.

Integrated feature	Classifier	Acc (%)	Sen (%)	Spe (%)	F1-Score (%)	MCC	AUC
PSSM-RICLBP+QLC + ESM2	DNN	90.66	89.91	91.43	90.62	0.8142	0.9678
	CNN	91.35	89.34	93.39	91.20	0.8284	0.9723
	BiLSTM	91.39	89.58	93.23	91.28	0.8290	0.9720
	GRU	90.91	89.42	92.41	90.79	0.8197	0.9717
	BiGRU	91.84	91.84	91.85	91.90	0.8378	0.9734
PSSM-RICLBP+QLC + ProtT5	DNN	91.15	90.31	92.01	91.09	0.8239	0.9685
	CNN	92.09	92.57	91.60	92.17	0.8424	0.9740
	BiLSTM	91.68	90.79	92.58	91.64	0.8345	0.9758
	GRU	91.88	90.31	93.48	91.80	0.8388	0.9757
	BiGRU	91.56	89.90	93.15	91.45	0.8322	0.9740
PSSM-RICLBP+QLC + ESM2 + ProtT5	DNN	89.53	88.37	90.70	89.43	0.7917	0.9597
	CNN	90.91	89.01	92.82	90.76	0.8194	0.9723
	BiLSTM	91.56	9047	92.66	91.50	0.8320	0.9749
	GRU	90.83	89.26	92.41	90.71	0.8178	0.9713
	BiGRU	91.84	90.47	93.23	91.76	0.8378	0.9772

Revisiting Table 1 and comparing the results of individual features with hybrid features in Table 3, we can see that the performance of all models improved. Specifically, individual ESM2 achieved the best performance on BiGRU with Acc = 91.31%, F1-Score = 91.28%, MCC = 0.8273, and AUC = 0.9734, while the hybrid PSSM-RICLBP+QLC + ProtT5 achieved the best Acc = 92.09, F1-Score = 92.17, MCC = 0.8424, and AUC = 0.9740 on the CNN, which is improved by 0.78%, 0.89%, 1.51%, and 0.06%, respectively. These results experimentally verified that integrated features can further improve the performance of the models. By further observing Table 3, we can see that the BiGRU which was the best performing model on the individual features, achieved consistent performance across three hybrid feature sets, among which it achieved the best comparable performance on PSSM-RICLBP+QLC + ESM2 and PSSM-RICLBP+QLC + ESM2 + ProtT5 with Acc of 91.81 and 91.84, F1-Score of 91.90 and 91.76, MCC of 0.8378 and 0.8378, and AUC of 0.9734 and 0.9772, respectively. Overall, all the hybrid feature combinations achieved better and competitive performance than the individual feature sets on all the classification models using 10-fold CV.

Although the results are improved over individual feature sets, they still lack substantial improvement. The main reasons for this behavior are (a) hybrid features often contain redundant and irrelevant information and (b) the dimension of the hybrid feature sets is relatively larger, which further restricts them from drastic improvements over individual features. An FS approach is important to solve these issues in hybrid feature sets. Therefore, in the following subsection, we will employ the FS method to the above three hybrid combinations.

### Feature selection can improve predictive performance

As discussed in the previous subsection, FS is vital for selecting the optimal features to achieve efficient generalized predictive performance. Therefore, the SBWOA FS technique was applied to three hybrid combinations of feature sets. Specifically, the dimensions of PSSM-RICLBP+QLC + ESM2, PSSM-RICLBP+QLC + ProtT5, and PSSM-RICLBP+QLC + ESM2 + ProtT5 were 1699, 1443, and 2723, respectively. Once the FS was employed, the SBWOA selected the optimal feature set of 257, 365, and 548 dimensions for (PSSM-RICLBP+QLC + ESM2)^SBWOA^, (PSSM-RICLBP+QLC + ProtT5)^SBWOA^, and (PSSM-RICLBP+QLC + ESM2 + ProtT5)^SBWOA^, respectively. Each optimal feature set was then fed into various learning models and the experimental outcomes were recorded in Table 4.

**Table 4:** Performance comparison of integrated features after SBWOA optimization using IR2476 training dataset.

Optimal feature	Classifier	Acc (%)	Sen (%)	Spe (%)	F1-Score (%)	MCC	AUC
(PSSM-RICLBP+QLC + ESM2)^SBWOA^	DNN	94.62	94.42	94.82	94.61	0.8928	0.9905
	CNN	93.90	94.02	93.77	93.89	0.8786	0.9865
	BiLSTM	94.70	95.07	94.34	94.71	0.8928	0.9897
	GRU	94.34	94.42	94.26	94.34	0.8874	0.9905
	BiGRU	94.91	94.42	95.39	94.88	0.8987	0.9906
(PSSM-RICLBP+QLC + ProtT5)^SBWOA^	DNN	94.79	94.66	94.90	94.78	0.8961	0.9919
	CNN	94.34	94.50	94.18	94.36	0.8872	0.9887
	BiLSTM	94.99	94.91	95.97	94.98	0.8999	0.9924
	GRU	95.03	94.99	95.07	95.02	0.9009	0.9920
	BiGRU	95.27	94.58	95.96	95.23	0.9057	0.9920
(PSSM-RICLBP+QLC + ESM2 + ProtT5)^SBWOA^	DNN	95.27	95.15	95.39	95.26	0.9056	0.9914
	CNN	94.42	94.26	94.58	94.40	0.8889	0.9881
	BiLSTM	95.07	95.31	94.82	95.08	0.9017	0.9920
	GRU	95.15	95.23	95.07	95.15	0.9033	0.9916
	BiGRU	95.96	95.55	95.36	95.93	0.9194	0.9924

We can see in Table 4 that after employing SBWOA FS, the results were significantly improved compared with the hybrid feature sets before employing SBWOA FS in Table 3. Specifically, the lowest performance achieved by the optimal feature set, i.e. (PSSM-RICLBP+QLC + ESM2) ^SBWOA^, was Acc = 93.90%, F1-Score = 93.89%, MCC = 0.8786, and AUC of 0.9865, which was improved by 1.81%, 1.72%, 3.62%, and 1.25%, respectively, compared with the highest performance achieved by original hybrid feature set, i.e. PSSM-RICLBP+QLC + ProtT5 in Table 3. Additionally, the dimensionality of these two features exhibits a substantial gap and training the models on the optimal feature set can significantly reduce the training burden, time, and resources, which again verifies the effectiveness of the FS method.

By comparing the three combinations of optimal feature sets in Table 4, we can easily conclude that the (PSSM-RICLBP+QLC + ESM2 +ProtT5) ^SBWOA^ obtained the best performance of 95.96%, 95.93%, 0.9194 and 0.9924 in terms of Acc, F1-Score, MCC and AUC, respectively, compared with the other two optimal feature sets. In addition, comparing all the classification models, the (PSSM-RICLBP+QLC +ESM2 + ProtT5) ^SBWOA^ achieved the highest performance on the BiGRU model. To further verify the model output, Fig. 6 illustrates the performance of the best optimal feature set, i.e. (PSSM-RICLBP+QLC + ESM2 + ProtT5) ^SBWOA^ on all the classification models, in terms of ROC and PR curves. By carefully examining the ROC in Fig. 6A and the PR curves in Fig. 6B, it is concluded that the BiGRU has a more robust performance with an AUC of 0.9924 and an AUPR of 0.9923.

**Figure 6:**
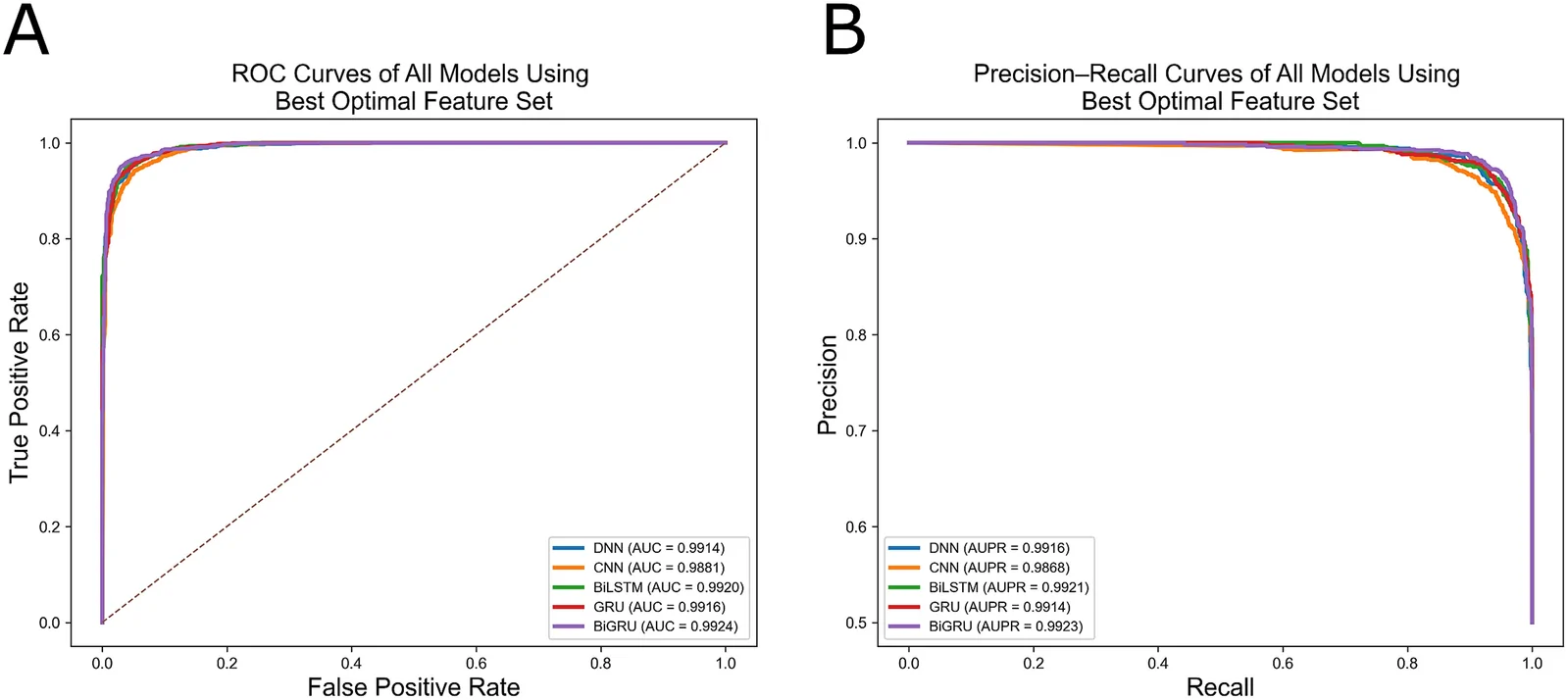
ROC and PR curves of best optimal feature set: (A) shows the ROC curves while (B) shows the PR curves of (PSSM-RICLBP+QLC + ESM2 + ProtT5) ^SBWOA^ on all the reported classification models.

Next, to further verify and show the performance of the hybrid feature set (PSSM-RICLBP+QLC + ESM2 + ProtT5) before and after applying SBWOA, the grouped bar chart is provided in Supplementary Figs S2 and S3 under Supplementary Text S5 in SI, where Supplementary Fig. S2 shows the comparison in terms of Acc, Sen, Spe, and F1-Score while Supplementary Fig. S3 shows the comparison in terms of MCC and AUC. By observing both Supplementary Figs S2 and S3, the Acc, F1-Score, MCC, and AUC, after applying SBWOA FS, were improved by 4.12%, 4.17%, 8.16%, and 1.63%, respectively, which shows the effectiveness of SBWOA. Together, the (PSSM-RICLBP+QLC + ESM2 + ProtT5) ^SBWOA^ achieved the highest performance and thus, in this study, the (PSSM-RICLBP+QLC + ESM2 +ProtT5) ^SBWOA^ is selected as the final optimal feature set to train the DeepIR-Pred model.

### Model generalization capability on unseen data

To further demonstrate the generalization ability of the proposed model, in this section, we performed extensive experiments on the independent dataset. It is noteworthy that this dataset was not used during the training process. In addition, the training dataset was exclusively sent to SBWOA to select optimal features and the same selected feature indices were applied to obtain optimal features for the independent dataset. Once the model was trained on the training dataset IR2476, the independent dataset IR274 was used to measure the generalization ability. The performance outcomes for each model were recorded in Table 5.

**Table 5:** Performance comparison on the independent IR274 test set.

Dataset	Classifier	Acc (%)	Sen (%)	Spe (%)	F1-Score (%)	MCC	AUC
Independent dataset (IR274)	DNN	92.33	89.79	94.89	92.13	0.8478	0.9805
	CNN	91.97	89.78	94.16	91.79	0.8402	0.9772
	BiLSTM	91.24	87.60	94.89	90.90	0.8270	0.9750
	GRU	90.87	87.59	94.16	90.56	0.8192	0.9748
	BiGRU	93.82	92.40	95.10	93.82	0.8764	0.9853

In Table 5, all the classification models obtained consistent performance, with BiGRU achieving the best results of Acc = 93.82, F1-Score = 93.82, MCC = 0.8764, and AUC = 0.9853. The second-best model was the DNN model with Acc, F1-Score, MCC, and AUC of 92.33, 92.13, 0.8478, and 0.9805, respectively. In addition, to further show the performance of the models, we also calculated the threshold-independent ROC and PR curves and the output plots are illustrated in Fig. 7A and B. From Fig. 7A, which shows the ROC curves for all the models on the independent dataset, we can see that the overall performance of the classification models is consistent with BiGRU as the standout. Similarly, for the PR curves in Fig. 7B, BiGRU also attained an AUPR of 0.9845, followed by DNN with an AUPR of 0.9812. To facilitate a clear performance analysis, a heatmap representation was also generated, as shown in Fig. 8. Together, from Table 5, Fig. 7A and B, and Fig. 8, we can clearly conclude that the proposed model achieved robust performance on the independent dataset, which shows the model’s effectiveness in terms of generalization capability. Since the BiGRU performed better than the other reported classification models, the final DeepIR-Pred model is trained on the BiGRU. The optimal hyperparameter settings for training the final model are provided in Supplementary Table S2 in Supplementary Text S6 of the SI.

**Figure 7:**
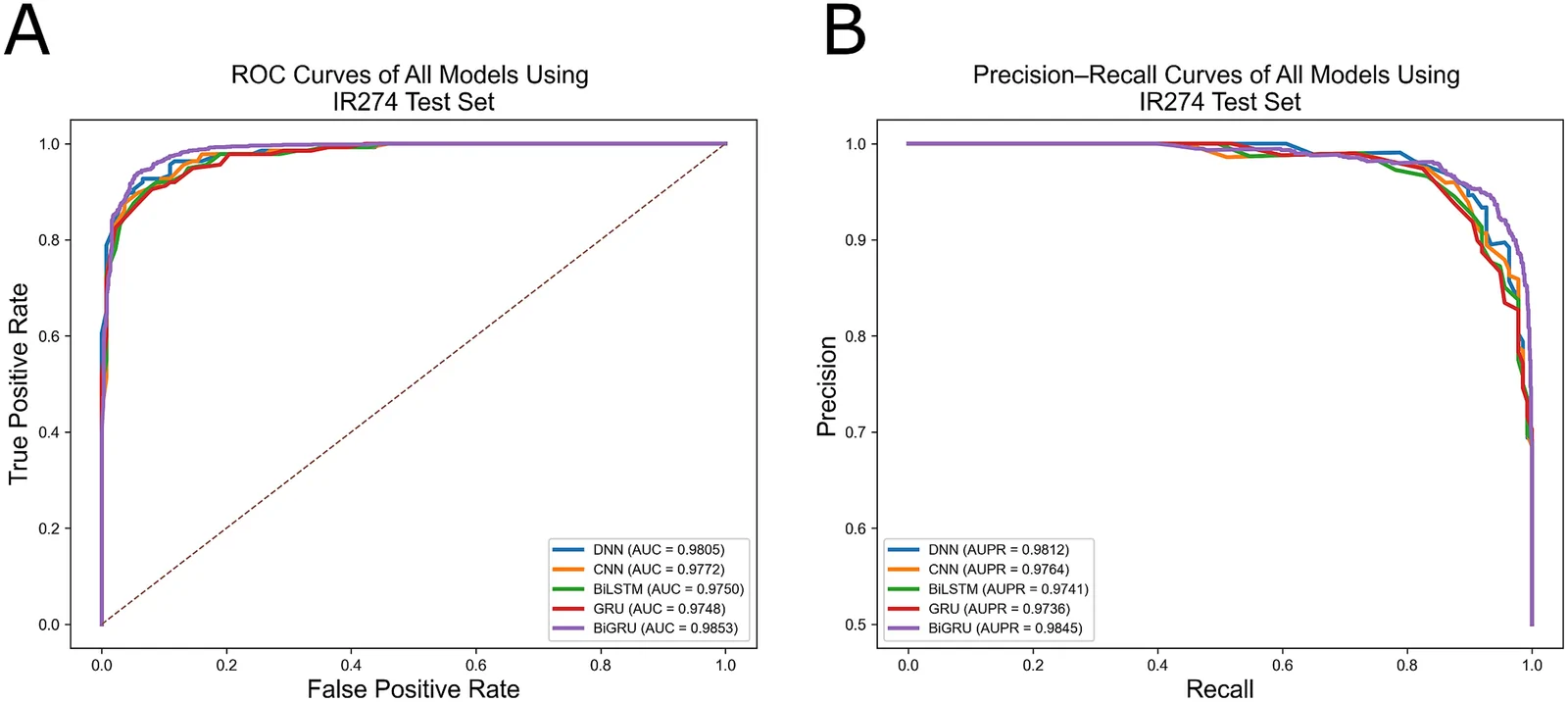
ROC and PR curves of all models using the independent dataset: (a) shows the ROC curves while (b) shows the PR curves.

**Figure 8:**
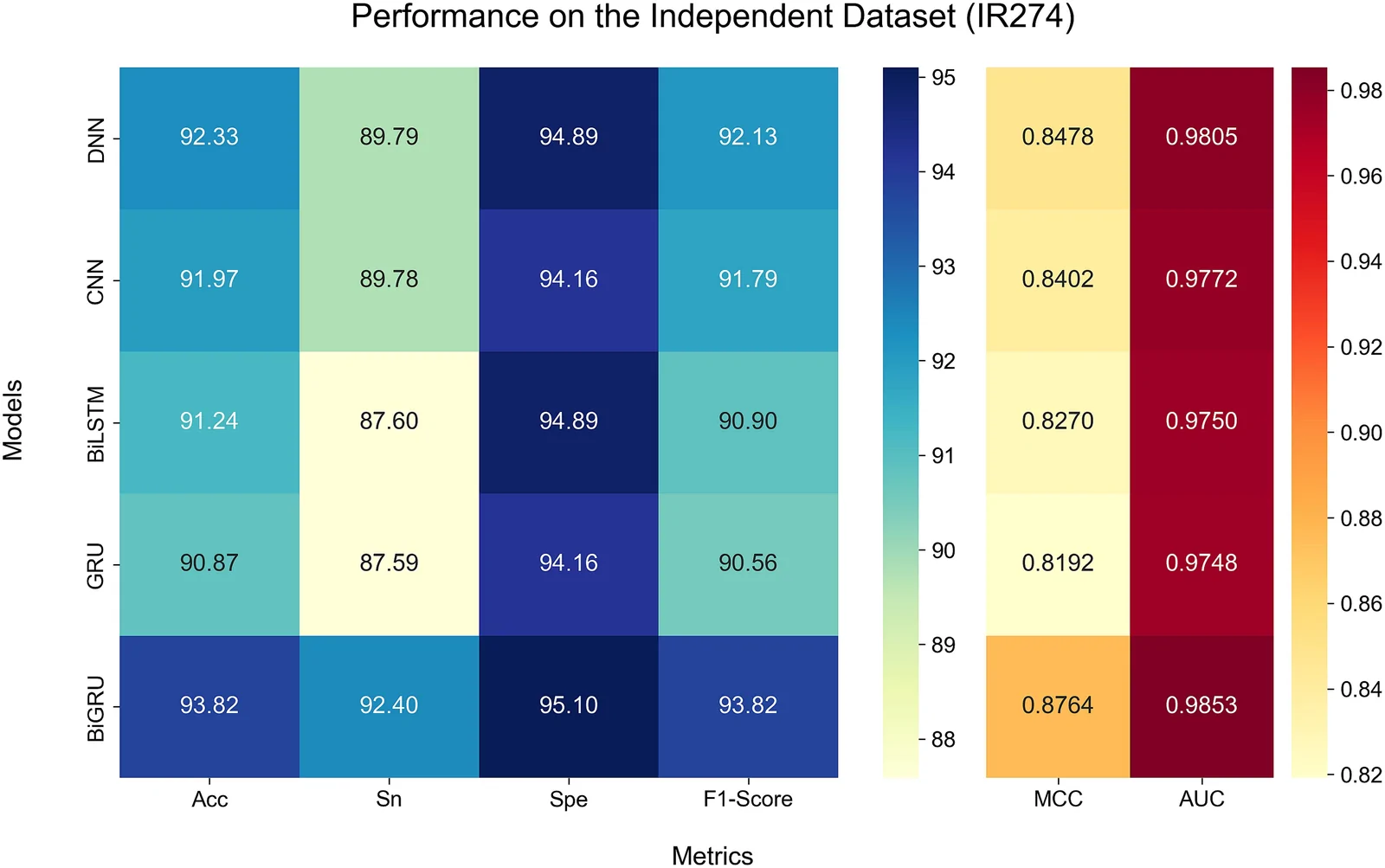
Performance comparison of models on the independent dataset using heatmap.

Table 6 is provided to easily grasp the performance of the final DeepIR-Pred on both the training dataset with 10-fold CV and the independent dataset where all samples were unseen during model training. The performance on both the training and independent datasets validates the real-world applicability of DeepIR-Pred for distinguishing IR proteins from non-IR proteins.

**Table 6:** Comparison of the final model performance on both training (using 10-fold CV) and independent datasets.

Proposed model	Dataset	Acc (%)	Sen (%)	Spe (%)	F1-Score (%)	MCC	AUC
DeepIR-Pred	Training (IR2476)	95.96	95.55	95.36	95.93	0.9194	0.9924
	Independent (IR274)	93.82	92.40	95.10	93.82	0.8764	0.9853

It is worth noting that the benchmark datasets were constructed using the latest release of UniProt, ensuring that recently discovered IR proteins were included. Given the strong prediction performance on an independent dataset, DeepIR-Pred is expected to generalize well to newly annotated IR proteins. However, future UniProt releases may include proteins with increased sequence diversity or distinct patterns that are underrepresented in the current training sequences. Consequently, periodically updating the training dataset and retraining DeepIR-Pred will further improve its robustness, generalization, and long-term applicability.

### DeepIR-Pred interpretability and feature analysis

To comprehensively understand the decision-making behavior and feature representation learning ability of DeepIR-Pred, several interpretability and visualization analyses were carried out. SHapley Additive exPlanation (SHAP) [44] analysis was employed to examine the significance and contribution of individual feature sets to model predictions. Figure 9 shows the SHAP summary analysis of the top 10 most informative features. The analysis showed that all four extracted feature sets (PSSM-RICLBP, QLC, ESM2, and ProtT5) contributed considerably to the discriminative capability of DeepIR-Pred. In particular, ProtT5_58, ESM2_251, and ESM2_68 exhibited the highest impact on the model predictions, suggesting that pLM-based embeddings were highly effective in discriminating IR and non-IR proteins. Several of the proposed features (PSSM-RICLBP_11 and PSSM-RICLBP_53) also showed a strong contribution to model performance, indicating that the transformed image-based evolutionary information remained a key complementary descriptor. In addition, the coexistence of positive and negative SHAP values across several features indicates that they contribute according to feature values and sequence characteristics. The feature group importance is provided in Supplementary Fig. S4 under Supplementary Text S7 in SI.

**Figure 9:**
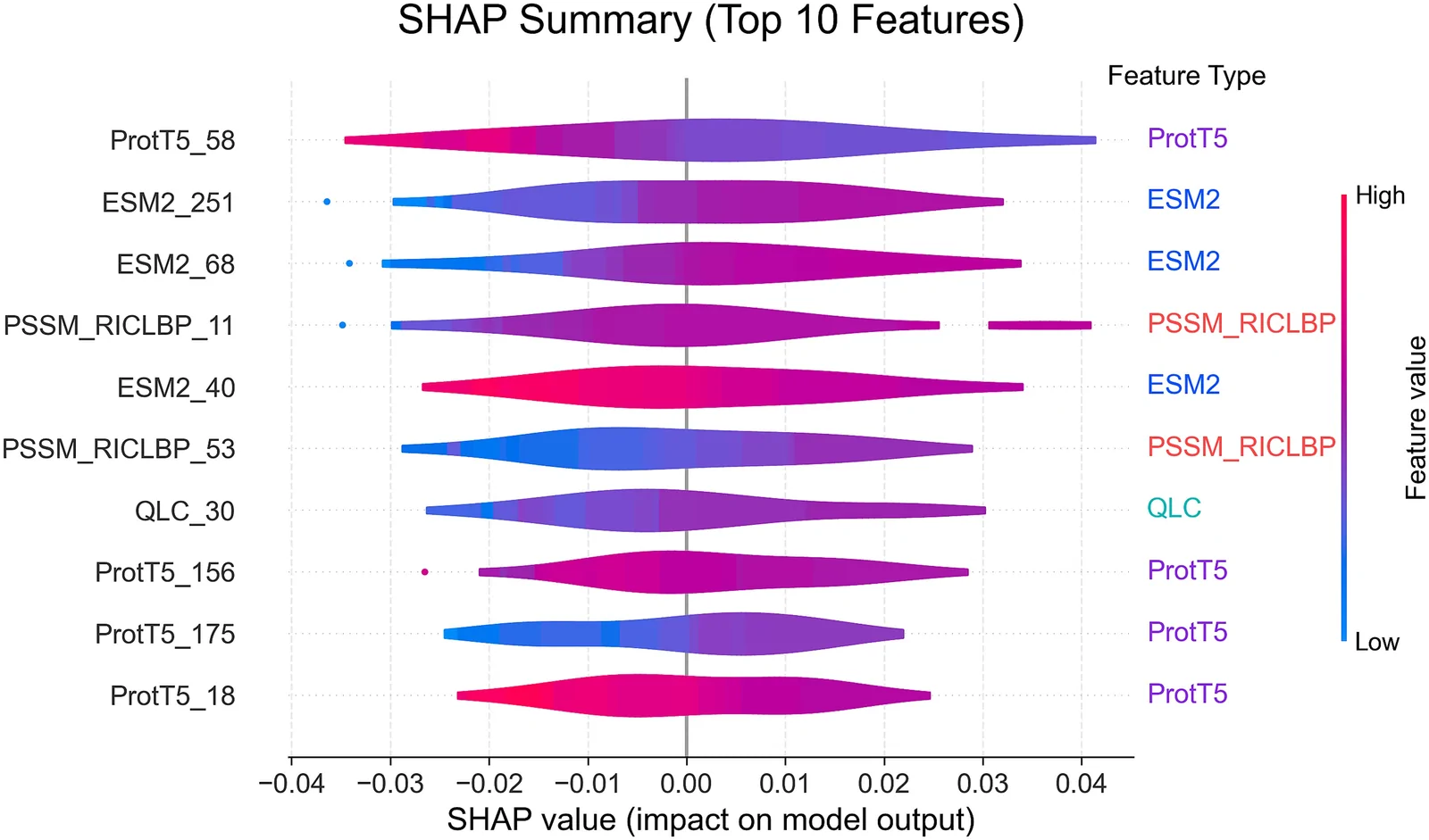
SHAP analysis showing top 10 most informative features.

Similarly, Local Interpretable Model-agnostic Explanations (LIME) [45] analysis was performed to provide a local interpretation of instance-based predictions as shown in Fig. 10. The sample was randomly chosen from the independent IR274 dataset. The model correctly predicted the sample as IR with a prediction probability of 1.0 while 0.0 for the non-IR, suggesting a highly confident prediction. LIME analysis further identified several features from all four feature sets that contributed positively to IR prediction. The majority of features were once again derived from pLMs embeddings, while the PSSM-RICLBP_27 further emphasized the importance of transformed image-based representations from PSSMs in IR identification. It is worth noting that only one feature, ProtT5_128, contributed negatively and supported non-IR prediction. The LIME analysis was also performed on an instance selected from the non-IR samples and the output report is provided in Supplementary Fig. S5 under Supplementary Text S8 in SI.

**Figure 10:**
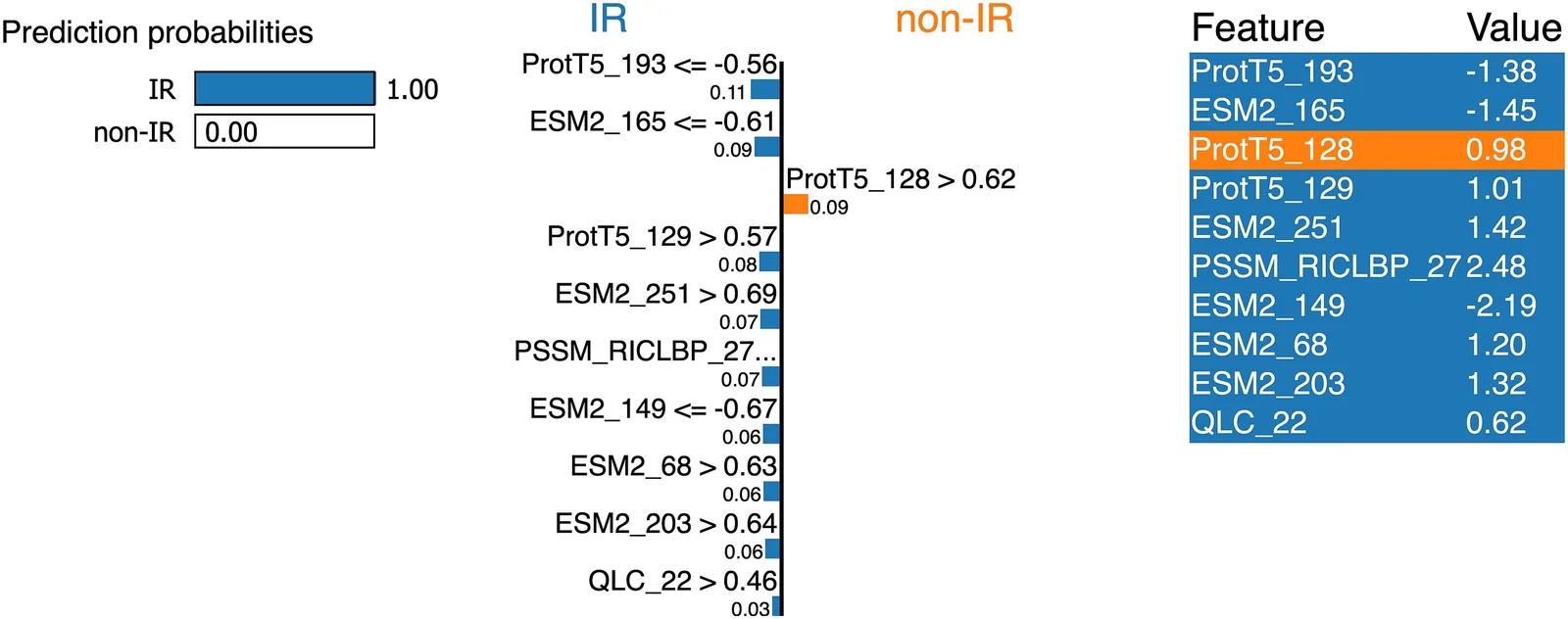
LIME analysis of DeepIR-Pred using IR sample from independent dataset.

Additionally, UMAP-based visualization [46] was performed on features from both the training and testing datasets to examine the representation learning capability of DeepIR-Pred. Figure 11A–C illustrates the UMAP visualization of feature representation from various stages of the DeepIR-Pred architecture using the independent IR274 dataset. In Fig. 11A, the input feature space, which is the optimal feature set from SBWOA, exhibits partial overlap between IR and non-IR protein samples, suggesting that although the optimal features contained discriminative information, partial overlaps were still present. Following the visualization plot of transformed features extracted from the BiGRU’s last bidirectional layer (Fig. 11B), the IR and non-IR proteins formed clear clustering patterns with very small overlap. The penultimate-layer embeddings, as shown in Fig. 11C, further retained class-specific structural patterns while yielding a smooth and organized feature distribution. Overall, the UMAP analysis suggests that DeepIR-Pred effectively transformed input features into highly informative and discriminative representations, which improved the separability of IR from non-IR in the learned feature space. Supplementary Figure S6 under Supplementary Text S9 in the SI reports the learning behavior on the training IR2476 dataset.

**Figure 11:**
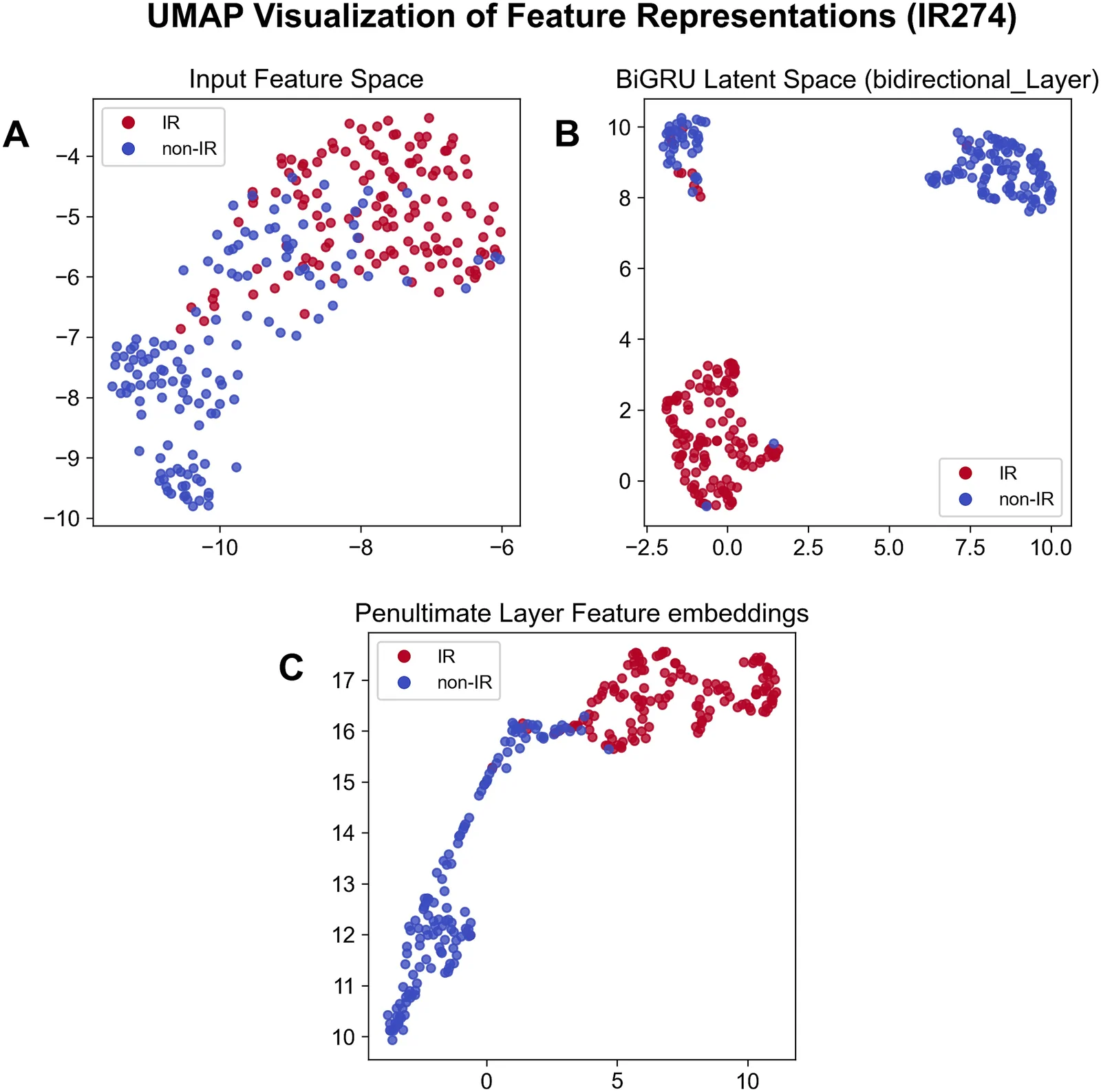
UMAP visualization of features extracted from different stages of DeepIR-Pred using independent dataset.

## Conclusion

In this work, a novel and efficient computational-based prediction model, DeepIR-Pred, was developed to effectively solve the problem of distinguishing IR proteins from non-IR proteins. To achieve this, first, a new handcrafted feature extraction method, PSSM-RICLBP, was proposed and employed together with the QLC, ESM2, and ProtT5 to effectively represent the protein samples in numerical representation. These feature extraction methods laid a strong foundation by capturing evolutionary, physicochemical, and deep contextual information in protein samples. Second, all extracted features were integrated to obtain a powerful hybrid feature set, containing all the information captured by individual feature sets. To reduce the dimensionality of the hybrid feature set and obtain a more robust optimal feature set, the SBWOA FS method was utilized. Third, various deep learning models were trained and tested, among which the designed BiGRU model achieved the best performance. Finally, the BiGRU model was used to train the proposed DeepIR-Pred. The robust and generalization capability using 10-fold CV on the IR2476 and independent testing on the IR274 proved that the proposed DeepIR-Pred can be adopted into real-world applications, such as research academia and drug discovery. The outstanding performance of the proposed DeepIR-Pred is due to the rigorous construction of training and independent datasets, the use of SBWOA to obtain a hybrid optimal feature set that includes all the evolutionary, physicochemical, and deep contextual information captured by individual features hidden in protein samples, and the selection of a deep recurrent learning model. The Explainable AI and UMAP visualizations further validated the model’s interpretability and the discriminative capabilities of the learned representations.

We believe that the current DeepIR-Pred model is robust and has robust generalization capability; however, in the future, we will investigate additional feature extraction and classification methods to further enhance the IR prediction. Advanced feature integration and FS methods will be developed to strengthen the optimal feature set. Evaluating the proposed model with more challenging datasets that explicitly include highly homologous receptor tyrosine kinase families and other demanding protein datasets is another key consideration for the future. Additionally, a user-friendly web server is also a consideration for future IR-based prediction models.

**Key Points**

- The study developed DeepIR-Pred, a biologically aware deep learning model for the identification of insulin receptors from sequence information.
- Two rigorously curated non-redundant benchmark datasets, IR2476 and IR274, were constructed to support robust training and generalization of the proposed model.
- The proposed novel transformed image-based descriptor (Position-Specific Scoring Matrix–rotation-invariant co-occurrence among adjacent local binary patterns) was integrated with qualitative characteristics and protein language model embeddings to capture biological patterns and information in insulin receptor (IR) sequences.
- The metaheuristic-based feature optimization and deep recurrent learning architecture exhibited robust performance and generalization capability on unseen samples.
- Extensive interpretability and feature visualization analyses using explainable AI and UMAP techniques investigated discriminative feature representations associated with IR proteins.

## Supplementary Material

Supplementary_materials_bbag523

## Data Availability

All data generated or analyzed during this study are publicly available at https://github.com/MateeullahKhan/DeepIR-Pred.
