# Supplementary material for: DeepIR-Pred: identification of insulin receptors using metaheuristic optimization of biologically informed multi-view features with deep recurrent learning
Source: Brief Bioinform. 2026 Sep 28;27(5):bbag523. doi: 10.1093/bib/bbag523 (PMC13617576; doi:10.1093/bib/bbag523)
Supplement: Supplementary file 1 [file supplementary_materials_bbag523.docx]

**Supporting Information**

**DeepIR-Pred: Identification of Insulin Receptors using Metaheuristic Optimization of Biologically Informed Multi-View Features with Deep Recurrent Learning**

Matee Ullah^1, 2^, Zhen Li^1^, Yixiao Zhai^1, 3^, Changwei Lv^1^, Kashif Ahmad Khan^4^, Quan Zou^5, 6, *^, Xu Tan^1, *^

^1^School of Artificial Intelligence, Shenzhen University of Information Technology, Shenzhen, 518055, China

^2^School of Life Science and Technology, University of Electronic Science and Technology of China, Chengdu, Sichuan 610054, China

^3^School of Mechanical Engineering, Tongji University, Shanghai, 200092, China

^4^Department of Computer Science, Abdul Wali Khan University Mardan, Mardan 23200, Pakistan

^5^Institute of Fundamental and Frontier Sciences, University of Electronic Science and Technology of China, Chengdu, Sichuan 610054, China

^6^Yangtze Delta Region Institute (Quzhou), University of Electronic Science and Technology of China, Quzhou, Zhejiang 324003, China

*To whom correspondence should be addressed.

**Text S1. Compositional analysis of Training Samples**

The sequence compositional analysis of the training IR2476 dataset for both IR and non-IR are presented in **Figure S1**. The Y-axis shows the amino acid’s percentage occurrence in both IR and non-IR sequences while the X-axis represents the 20 amino acids. overall, the IR and non-IR share the similar composition with Ala (A), (Glu) E, (Leu) L, (Ser) S and (Val) V amino acid residues have high occurrence percentage. However, there are also some differences such as the residues (Pro) P, (Gln) Q, Ser, and (Thr) T occurrence percentage is higher in IR sequences whereas Ala, (Asp) D, (Gly) G, (Ile) I, (Lys) K and Val appeared more dominantly in non-IR sequences. Despite these variations, Leu, (Met) M, (Asn) N appeared nearly in identical proportion in IR and non-IR sequences which indicates limited discriminative ability. Overall, the variations are moderate but suggests that the composition of amino acid residues contributes to discriminate IR and non-IR samples.

**Figure S1**. Distribution of Amino Acids in the Training Dataset.

# **Text S2. Qualitative Characteristics for Amino Acid Residues**

**Table S1.** Qualitative Characteristics for amino acid residues based on three distinct groups.

| Physiochemical Attributes | Group 1 | Group 2 | Group 3 |
| --- | --- | --- | --- |
| Hydrophobicity | Polar | Neutral | Hydrophobicity |
|  | R,K,E,D,Q,N | G,A,S,T,P,H,Y | C,L,V,M,F,W |
| Normalized van der waals volume | 0-2.78 | 2.94-4.0 | 4.03-8.08 |
|  | G,A,S,T,P,D | N,V,E,C,Q,I,L | M,H,K,F,R,Y,W |
| Polarity | 4.9-6.2 | 8.0-9.2 | 10.4-13.0 |
|  | L,I,F,W,C,M,V,X | P,A,T,G,S | H,Q,R,K,N,E,D |
| Polarizability | 0-1.08 | 0.128-0.186 | 0.219-0.409 |
|  | G,A,S,D,T | C,P,N,E,Q,I,L | K,M,H,F,R,Y,W |
| Charge | Positive | Neutral | Negative |
|  | K,R | A,N,C,Q,GH,I,L,M,F,P,S,T,W,Y,V | D,E |
| Secondary structure | Helix | Strand | Coil |
|  | E,A,L,M,Q,KR,H | V,I,TC,W,F,T | G,N,P,S,D |
| Solvent-accessible | Buried | Exposed | Intermediate |
|  | A,L,FC,G,I,V,W | R,K,QE,N,D | M,S,PT,H,,Y |

# **Text S3. Mathematical Notations for Performance measures**

Following are the mathematical notations for the Accuracy (*Acc*), Sensitivity (*Sen*), Specificity (*Spe*) and the Matthew correlation coefficient (*MCC*).

$$Acc=\frac{TP+TN}{TP+FP+TN+FN}$$

$$Sen=\frac{TP}{TP+FN}$$

$$Spe=\frac{TN}{TN+FP}$$

$$F1-Score=\frac{\text{Pr + Sen}}{\text{Pr + Sen}}$$

where *Pr* means Precision, which can be mathematically represented as:

$$\text{Pr}=\frac{TP}{TP+FP}$$

$$MCC=\frac{TP\cdot TN-FP\cdot FN}{\sqrt{(TP+FP)\cdot(TP+FN)\cdot(TN+FP)\cdot(TN+FN)}}$$

where $TP$ represents true IR proteins that are correctly predicted by the model as positive instances, $TN$ represents true non-IR proteins that are correctly classified by the model as negative instances, $FN$ represents IR proteins that are incorrectly predicted as non-IR proteins and $FP$ represents non-IR proteins that are incorrectly predicted as IR proteins.

# **Text S4. Software and Hardware Configuration**

All experiments were tested in Python 3.11.13 utilizing TensorFlow 2.16.2, Keras 3.12, NumPy 1.26.4, Pandas 2.3.3, PyTorch 2.9.1 and Scikit-learn 1.8.0. The proposed DeepIR-Pred was trained and evaluated on a PC equipped with an Apple M1 Pro processor, 16GB of unified memory with macOS Tahoe as running OS. The complete data, source code and implementation details are publicly available through the DeepIR-Pred GitHub repository provided in the manuscript.

**Text S5. Performance comparison Before and after applying SBWOA**

**Figure S2**. Performance comparison of (PSSM-RICLBP+QLC+ESM2+ProtT5) before and after applying SBWOA feature selection in terms of Acc, Sen, Spe and F1-Score.

# **Text S6. Optimal Hyperparameters Used for Training the Final Model**

**Figure S3**. Performance comparison of (PSSM-RICLBP+QLC+ESM2+ProtT5) before and after applying SBWOA feature selection in terms of MCC and AUC.

The following Table S2 provides the optimal hyperparameters used for training the DeepIR-Pred model.

| **Table S2.** Hyperparameter settings of the DeepIR-Pred model. | |
| --- | --- |
| **Layers/Hyperparameters** | **Value** |
| BiGRU Layers | 256, 128 |
| Return Sequences | Layer 1 = True, Layer 2 = False |
| Dropout | 0.4, 0.3, 0.2 |
| Recurrent Dropout | 0.4, 0.3 |
| Dense hidden layer | 64 |
| Activation Function | ReLu, Sigmoid |
| Layer Normalization | Epsilon = 1×10⁻⁶ |
| Optimizer | Adam |
| Learning rate | 0.001 - 1×10⁻⁶ with factor = 0.1 |
| Batch Size | 16 |
| Loss function | Binary Cross-Entropy |

# **Text S7. SHAP Analysis on Feature Group Importance**

**Figure S4** shows the SHAP-based feature group importance analysis for the four feature sets used in DeepIR-Pred i.e. PSSM-RICLBP, QLC, ESM2 and ProtT5. Among all the four feature groups, ESM2 and ProtT5 embeddings revealed the highest average SHAP values, suggesting that transformer-based pLMs representations contributed substantially to the predictive capability of the proposed DeepIR-Pred model. In contrast, PSSM_RICLBP was the third best representation while QLC descriptor showed comparatively lower but still meaningful contribution to the prediction performance, indicating that both evolutionary and sequence composition-based physicochemical information provided complementary discriminative insights for identifying IR proteins.

**Figure S4**. SHAP analysis showing the feature group importance.

# **Text S8. LIME Analysis of the non-IR Sample**

**Figure S5** shows the LIME analysis of DeepIR-Pred model using non-IR sample from independent dataset. a random sample was chosen from the non-IR proteins to show the analysis report. We can see that model predicted the sequence as non-IR proteins with prediction probability of 0.99 and 0.01 for the IR, shows the confidence level of the model. Most of the ProtT5 features were positive toward non-IR. Interestingly, the PSSM-RICLBP contributed positively when predicting both IR (reported in the manuscript) and non-IR proteins, suggesting they have high impact on distinguishing IR and non-IR.

Figure S5. LIME analysis of DeepIR-Pred using non-IR sample from independent dataset.

# **Text S9. UMAP Analysis of the Features Using Training Dataset**

**Figure S6(A-C)** illustrates the UMAP visualization of feature representation from various stages of DeepIR-Pred architecture using the training IR2474 dataset. As discussed in the manuscript, the input feature space (**Figrue S6A**) after the SBWOA contains some discriminative information, however, there are still overlap between IR and non-IR protein samples. The latent feature space from the BiGRU last bidirectional layer showed tremendous clustering patterns with unnoticeable overlap (**Figure S6B**). Similarly, the penultimate layer embeddings (**Figure S6C**) increased the clustering distance drastically which once again proves the model effectively transformed input features into highly informative and discriminative representations, drastically improving the prediction of IR and non-IR in the learned feature space.

**Figure S6**. UMAP visualization of features extracted from different stages of DeepIR-Pred using training dataset.
